# Autophagy: Multiple Mechanisms to Protect Skin from Ultraviolet Radiation-Driven Photoaging

**DOI:** 10.1155/2019/8135985

**Published:** 2019-12-13

**Authors:** Mei Wang, Pourzand Charareh, Xia Lei, Julia Li Zhong

**Affiliations:** ^1^National Innovation and Attracting Talents “111” Base, Key Laboratory of Biorheological Science and Technology, Ministry of Education, Chongqing University, Chongqing 400044, China; ^2^Department of Dermatology, Daping Hospital, Army Medical University, Chongqing 400042, China; ^3^Department of Pharmacy and Pharmacology, University of Bath, Bath BA2 7AY, UK

## Abstract

Autophagy is an essential cellular process that maintains balanced cell life. Restriction in autophagy may induce degenerative changes in humans. Natural or pathological aging of susceptible tissues has been linked with reduced autophagic activity. Skin photoaging is an example of such pathological condition caused by ambient solar UV radiation exposure. The UV-induced production of reaction oxygen species (ROS) has been linked to the promotion and progression of the photoaging process in exposed tissues. Accordingly, it has been suggested that autophagy is capable of delaying the skin photoaging process caused by solar ultraviolet (UV), although the underlying mechanism is still under debate. This review highlights several plausible mechanisms by which UV-induced ROS activates the cellular signaling pathways and modulates the autophagy. More specifically, the UV-mediated regulation of autophagy and age-related transcription factors is discussed to pinpoint the contribution of autophagy to antiphotoaging effects in the skin. The outcome of this review will provide insights into design intervention strategies for delaying the phenomenon of sunlight-induced photodamage, photoaging, and other aging-related chronic diseases based on factors that activate the autophagy process in the skin.

## 1. Introduction

Autophagy is a vital homeostatic cellular process of either clearing surplus or damaged cell components notably lipids and proteins or recycling the content of the cells' cytoplasm to promote cell survival and adaptive responses during starvation and other oxidative and/or genotoxic stress conditions. Autophagy may also become a means of supplying nutrients to maintain a high cellular proliferation rate when needed [1]. Genotoxic stress usually occurs by a series of environmental and pharmacological agents, notably by solar ultraviolet (UV) radiation. It has been suggested that the induction of autophagy under these conditions is to try to alleviate the effects of oxidative DNA damage [2]. All UV components of sunlight, i.e., UVA (320-400 nm), UVB (280-320 nm), and UVC (100-280 nm), are capable of both causing DNA damage and inducing autophagy. Moreover, UV radiation of sunlight is capable of regulating a number of autophagy-linked genes [3–6]. Nevertheless, the mechanisms underlying these processes have not yet been fully elucidated. It is known that loss of autophagy leads to both photodamage and the initiation of photoaging in UV-exposed skin. Autophagy restriction may also induce several skin-related chronic disorders as well as skin cancer. This review will focus on critically appraising the cellular mechanisms suggested for the antiphotoaging action of the autophagy machinery in the skin cells induced by solar UV radiation.

### 1.1. Photoaging Mediated by UV Radiation

Skin aging is a highly complex and coordinated biological event comprising natural aging and solar radiation-mediated photoaging. The former process occurs naturally and results from slow tissue degeneration [7], while the latter occurs due to the accumulation of unavoidable chronic sun exposures in daily life. Once photoaging is initiated, collagen fibers are degraded, the skin becomes subsequently loose with wrinkles, and the pigmentation occurs on the skin due to abnormal proliferation of melanocytes. In addition, increasing matrix metalloproteinase (MMP) content leads to intracellular matrix degradation, inflammatory infiltrates, and vessel ectasia [8]. Prolonged UV exposure is considered to be a major cause of photoaging, leading to the abovementioned phenomena in the skin [9, 10].

The solar UV radiation that reaches the surface of the earth is composed of the longer UVA (320-400 nm) and the shorter UVB (280-320 nm) wavebands, respectively. Both radiations penetrate through the thick ozone layer and reach the biosphere. Long-wave UVA that comprises about 95% of solar terrestrial UV penetrates deeply into the dermal layer and even reaches the subcutaneous layer of the skin. Because of its oxidative nature, UVA is capable of damaging DNA and other biomolecules by ROS generation [11]. UVA-induced ROS formation has been implicated in the oxidation of DNA bases leading to signature DNA lesions such as 8-oxo-deoxyguanine (8-oxodG) which is a known potent mutagenic lesion [12]. The UVA component of sunlight has been considered the main cause of prominent changes in the dermal extracellular matrix (ECM) of the photoaged skin. UVB, which represents about 5% of terrestrial UV, can reach at least the epidermis as well as the upper dermis and can induce dermal changes through epidermis-to-dermis signaling [13]. The different biological effects of UVA and UVB are related to the type of biomolecules that they interact with.

**Figure 1:**
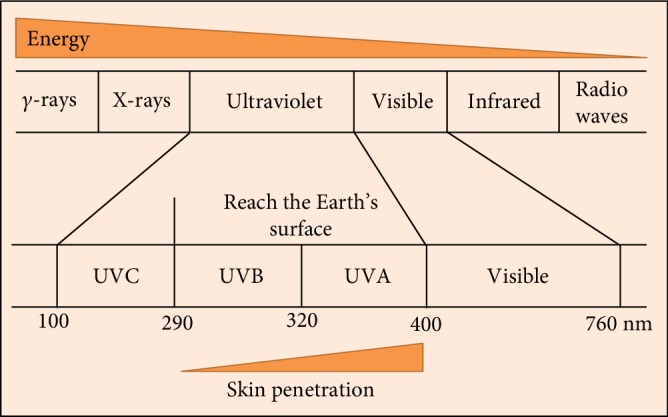
Ultraviolet (UV) as a component of the electromagnetic spectrum.

UVB radiation is primarily a DNA-damaging agent because it is directly absorbed by DNA and is known to cause cyclobutane pyrimidine dimers (CPDs) and 6-4 pyrimidine pyrimidone dimers (6-4PP) [14]. The unrepaired DNA lesions cause DNA mutation during cell division which may lead to the initiation of carcinogenesis [15]. Both nonenzymatic (i.e., glutathione and ascorbic acid) and enzymatic antioxidants such as superoxide dismutase (SOD), catalase (CAT), glutathione peroxidase (GPX), glutathione reductase, and thioredoxin reductase (TRX) are essential components of the skin defense against ROS-mediated damage. Nevertheless, the excess ROS production by UV radiation can overwhelm the endogenous antioxidant capacity of the skin. The latter justifies the use of exogenous antioxidants as photoprotectants to neutralize UV-mediated ROS production [16]. It has been suggested that both UVA and UVB initiate photoaging [17] by producing reactive oxygen species (ROS) that destroy cellular macromolecules such as proteins, lipids, and, more importantly, the genomic DNA [18]. UVA is known to be the oxidizing component of sunlight, and its damaging effect on biomolecules occurs indirectly, *via* the generation of ROS through its interaction with a variety of chromophores (e.g., porphyrins, bilirubin, and melanin) [19, 20]. Although ROS production by UVA can lead to DNA damage (Figure 1) [21], it has been recently shown that the proteome is one of the major targets of damage by UVA-induced ROS [22]. UVB, while it does have an oxidative component, induces specific lesions into DNA and damages proteins mostly by direct absorption [23]. UVC can cause damage to human health but is absorbed by the stratospheric ozone layer. It is a strong DNA-damaging agent, and this property has been exploited in building artificial UVC (207-222 nm) lamps that assess potent antimicrobial properties, and unlike the germicidal 254 nm UVC source, it is not harmful to the human skin due to its limited penetration distance of 207 nm light in biological samples (e.g., stratum corneum) compared with that of 254 nm light. This lamp source is an important agent against drug-resistant bacteria and airborne aerosolized viruses. Nevertheless, very low level of UVC can inactivate more than 95% of aerosolized H1N1 influenza virus [24, 25].

UVA and UVB have different effects on skin aging. Continuous UVB exposure can induce keratinocytes to produce more interleukin 1*α* (IL-1*α*), which initiates granulocyte-macrophage colony stimulatory factor (GM-CSF) secretion in an autocrine manner. Both IL-1*α* and GM-CSF molecules enter dermal tissues and activate fibroblasts to produce neprilysin. Neprilysin cleaves and disrupts the elastic fibrous network that wraps the fibroblasts. The combination of elastic and collagen fiber deficiency reduces skin elasticity, hence assisting in the formation of skin wrinkles. However, continuous exposure to UVA radiation leads the keratinocytes to produce GM-CSF in lower amounts than UVB exposure but activates dermal fibroblasts to the same extent as UVB radiation. Furthermore, UVA penetrates the dermal layers of the skin and immediately stimulates the expression of MMP-1 and secretion of IL-6, which mainly lead to sagging of the skin [26].

UV exposure is a major factor that induces photoaging. In particular, UVA irradiation induces ROS and subsequently promotes the oxidation of membrane lipids to form oxidized phospholipid-protein adducts. These oxidized adducts are cleared and degraded by autophagy to prevent cellular damage. Autophagy caused by environmental insults including UV radiation and the consequent generation of ROS appears necessary for survival and cell function as well as homeostasis and immune tolerance [27]. In keratinocytes, the basal level of autophagy augments considerably upon solar UV exposure, leading to epidermal thickening (hyperkeratosis) and then epidermal hyperplasia which acts as a protection against the penetration of UV rays to the skin [28]. The skin pigmentation has also been linked to autophagy, as it depends on the melanin from phagocytosed melanocytes engulfed by the keratinocytes [29]. Additionally, the promotion of lysosomal-dependent decomposition and reutilization of cytoplasmic inclusions in autophagy has recently been linked with aging [30, 31]. It has also been shown that decreased autophagy is linked with increased aging, while stimulating autophagy enhances antiaging effects [32–34]. Defects in autophagy have also been shown to cause severe inflammatory reaction in the skin, because of the activation of inflammasome activation, as well as the induction of ROS production by UVR and aberrant liberation of proinflammatory cytokine release [35]. In an attempt to gain an insight into these phenomena, it is necessary to review the recent findings in the mechanism underlying the autophagy process and machinery.

Within the electromagnetic spectrum, UV wavelengths are in the range of 100–400 nm. The UV components are further divided into several wavebands notably UVC, UVB, and UVA. UVC is absorbed by the stratospheric ozone layer, so only UVA and UVB can reach the surface of the Earth. With waveband increasing, tissue transmission is increased, while the energy is decreased. Thus, UVA can penetrate much deeper into the skin than UVB.

## 2. Autophagy Machinery

Autophagy is an essential, evolutionarily conserved lysosomal degradation pathway that eliminates protein aggregates and damaged organelles to control the quality of the cytoplasm [36]. It concludes in macroautophagy, chaperone-mediated autophagy, and microautophagy [36, 37].

Macroautophagy which is the main emphasis of the current review is a phenomenon that is predominantly involved in the degradation and clearance of nonliving proteins as well as the degradation of various subcellular organelles. It is highly conserved from unicellular organisms to human [38].

**Figure 2:**
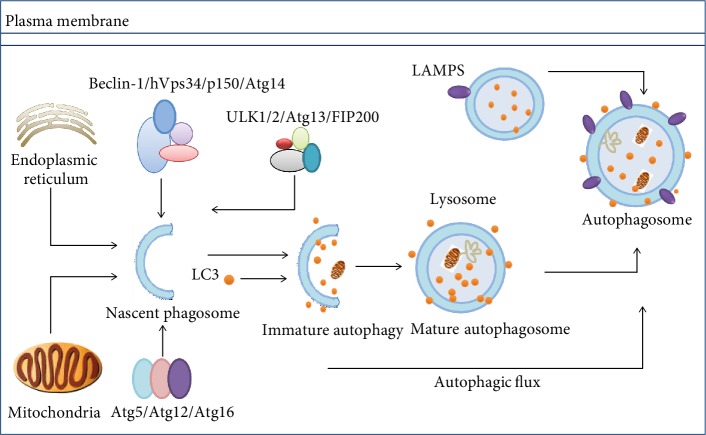
Overview of macroautophagy. Despite the occurrence of two membranes, bulk proteins and damaged organelles (ER and mitochondria) along with selective cargo within the cytoplasm are being engulfed, by small autophagosomes that fuse later to give full-sized autophagosomes. A number of autophagy-linked proteins and protein complexes are taking part in a highly coordinated procedure. Following fusion of autophagosomes with lysosomes, autolysosomes that decompose autophagic cargo to release recyclable biomolecules and macromolecules within the cytoplasm are formed (proteins involved: autophagy-linked enzymes Atg5, Atg12, Atg13, Atg14, and Atg16; autophagy-related proteins; Beclin-1 (Atg6); p150; (Vps15) serine/threonine-protein kinase. Vps34: class III phosphoinositide 3-kinase; Atg7: ubiquitin-E1-like enzyme; ULK1/2: unc-51-like autophagy-activating kinase 1/2; FIP200: family-interacting protein of 200 kD; LAMP2A: lysosomal-linked membrane protein 2A; LC3: microtubule-associated proteins 1A/1B light chain 3).

While autophagy in mammalian cells naturally occurs under normal circumstances, it can also be initiated under stress conditions. These include conditions such as starvation, infection by multiple types of pathogens, or exposure to pharmacological mediators, especially rapamycin. Moreover, despite its major role in restoring cellular homeostasis via the release of macromolecular nutrients, autophagy can promote the clearing of misfolded proteins as well as broken cellular inclusions and organelles (Figure 2). In macroautophagy, a double-membrane structure which is called the isolation membrane (or the phagophore) outgrows from the endoplasmic membrane (ER). It has been shown that the Golgi, the plasma membrane, and mitochondria also contribute to the growth of the budding autophagosome [39]. Macroautophagy occurs in three phases. These include the initiation of phagophores, then elongation phase, and the final degradation phase.

### 2.1. Initiation of Autophagy

Normally, the formation of autophagosomes initiates at the assembly points on phagophore assembly sites. Phagophore formation mainly demands the class III phosphoinositide 3-kinase (PI3K) Vps34, which functions in macromolecular complexes containing autophagy-linked proteins Atg6 (Beclin-1) and Atg14 as well as the Vps15 (p150) protein. Numerous proteins are involved in the early phases of autophagy notably autophagy-linked proteins Atg5, Atg12, and Atg16 as well as the focal adhesion kinase enzyme (FAK) and the 200 kDa family-interacting protein (FIP200), which constitutes the mammalian ortholog after conjugation with autophagy-linked proteins Atg1 (or ULK1) and Atg13 [40].

### 2.2. Elongation

Two ubiquitination-like reactions are associated with the elongation of phagophore membranes. Initially, ubiquitin-like Atg12 forms a complex with Atg5 by enzymatic conjugation to Atg7 (i.e., an E1 ubiquitin-activating-like enzyme) and Atg10 (i.e., an E2 ubiquitin-complexing mimicking enzyme). The autophagy-linked Atg5-Atg12 protein complex formation with Atg16L1 occurs through noncovalent binding. The complex then attaches to phagophores [41] and detaches from mature autophagosomes. During the second system of autophagosome formation, LC3 (MAP-LC3/Atg8/LC3) links to lipid phosphatidylethanolamine (PE) and gets stimulated by Atg7 (E1-like) and Atg3 (E2-like) to produce LC3-II [40]. Numerous LC3-positive autophagosomes that are randomly formed within the cytoplasm translocate along with the microtubules towards lysosomes in a dynein-dependent way and accumulate in the vicinity of the microtubule-organizing center (MTOC) adjacent to the nuclear membrane. Both lysosomes and autophagosomes are fused together by the action of two SNARE proteins, viz., Vti1B and VAMP8 [42].

### 2.3. Degradation of Autophagosomes

LC3-II promotes targeted degradation of long-lived and extensively utilized proteins, their aggregates, and injured or dead cellular organelles by interacting with adaptor proteins. P62 as a selective adaptor attaches to cargo proteins for the final degradation. In addition, p62 attachment may also occur through the LC3-interacting region (LIR) to link with LC3-II located on the outer side of the autophagosome membrane [43]. In addition, p62 is a target of specific substrates to the autophagosome and LC3-II and is used as a measure of autophagic flux [43]. The adaptor and cargo are degraded upon autophagosome-lysosome fusion. Autophagy products are recycled within the cytosol and help to restore important cellular processes following exposure to stressors and starvation.

## 3. Autophagy- and UV-Mediated Photoaging

The skin faces environmental UV exposure insult that causes oxidative damage to macromolecules [44]. Nuclear factor erythroid-derived 2-like 2 (Nrf2) activation/response is a centerpiece in resistance to oxidative stress, by upregulating antioxidant molecules and detoxifying enzymes to remove the ROS-mediated oxidative damage of cellular inclusions [3]. Autophagy is defined as an intracellular degradation phenomenon that is initiated to degrade oxidized lipids and metabolic wastes following UV exposure. It is therefore thought to decrease the progression of photoaging [45, 46].

In response to UVA irradiation or oxidized lipids, the Nrf2-driven antioxidant response activates the expression of cellular antioxidants and detoxifying enzymes such as heme oxygenase 1 (HO-1) [47–49]. In parallel to this process, proteins that were modified by ROS are cleared via proteasomal, autophagosomal, and lysosomal pathways in the cells [50]. Moreover, autophagosome LC3-II is formed through Atg7-dependent conjugation to a link between a PE (phosphatidylethanolamine) anchor and LC3 (the microtubule-linked protein 1 light chain 3). Cargo targeted for degradation is appropriated and destined for LC3-II via certain adaptor proteins, especially p62 (adaptor protein sequestosome 1 or SQSTM1). The complete structure is a spherical autophagosome with lysosomes that decompose various cargo proteins [51]. The involvement of both UVA- and UVA-oxidized PAPC- (1-palmitoyl-2-arachidonoyl-sn-glycero-3-phosphocholine-) mediated autophagy in epidermis-residing keratinocytes was reported. Various ROS induce rapid growth of high molecular weight protein masses comprising many different autophagy-related adaptor proteins, notably p62 (SQSTM1) in autophagy-deficient (Atg7-negative) keratinocytes [3]. Furthermore, autophagy is important in degrading proteins and modifying lipids following various environmental stresses, including UV exposure.

UVA-mediated ROS production mainly oxidizes phospholipids, which later forms oxidized phospholipid-protein adducts [3]. Autophagy then promotes the degradation of these metabolic adducts following UVA irradiation. Aging and aging-related diseases can be ravaged by minimizing these protein-based adducts and aggregates, crosslinking, and finally removing potentially toxic protein fragments from the cells. However, autophagy-mediated clearance of such waste declines over time [52], leading to an increase in oxidized phospholipid-protein adducts [53] and oxidized combination groups [54], which together accumulate and contribute to skin photoaging.

The mechanism of UV-induced autophagy still needs to be revealed because UVA irradiation induces autophagy that is impaired by treatment with the singlet oxygen quencher NaN3 [3, 55]. Similarly, UVB-induced autophagy is blocked by various antioxidants [56]. UV exposure promotes the formation of oxidized phospholipids, oxysterols, and cholesterol in keratinocytes [3, 57]. Moreover, 25-hydroxycholesterol (25-OH) is one of the oxidized lipids formed by UV exposure that is sufficient to activate autophagy in the skin keratinocytes and to perform a crucial function in inducing morphological changes and differentiation [57].

The inactivation of the essential autophagy-related genes significantly decreases the functions of sweat glands in aging mice [34]. It is suggested that ROS are critical cellular signal transducers and that solar UV light potentially generates ROS in the human skin [58]. Solar UV radiation modulates the activity of some autophagy/aging-linked genes [6]. It is imperative to highlight the types of stress regulation and the mechanism for both UVA and UVB irradiation. These factors are shown to regulate aging and autophagy as well as the relationship between aging and autophagy. In addition, UV radiation functions as the major environmental risk factor which causes skin cancer as about 50% of skin cancers are related to UV exposure [59]. In skin cancer, autophagy can be either oncogenic or tumor-suppressive, which mainly depends on the tumor cell type, stage of progression, carcinogenic context, etc. Autophagy acts as a tumor-suppressive mechanism by promoting ROS clearance, DNA repair, and oncogenic protein substrates [60, 61]. Alternatively, autophagy also facilitates tumor development by autophagy-mediated intracellular recycling that provides macromolecules with sustained cell proliferation. Additionally, autophagy has also been shown to be associated with UV-induced skin diseases, such as hyperpigmentation [62].

## 4. UV Radiation-Mediated Cell Signaling Pathway

**Figure 3:**
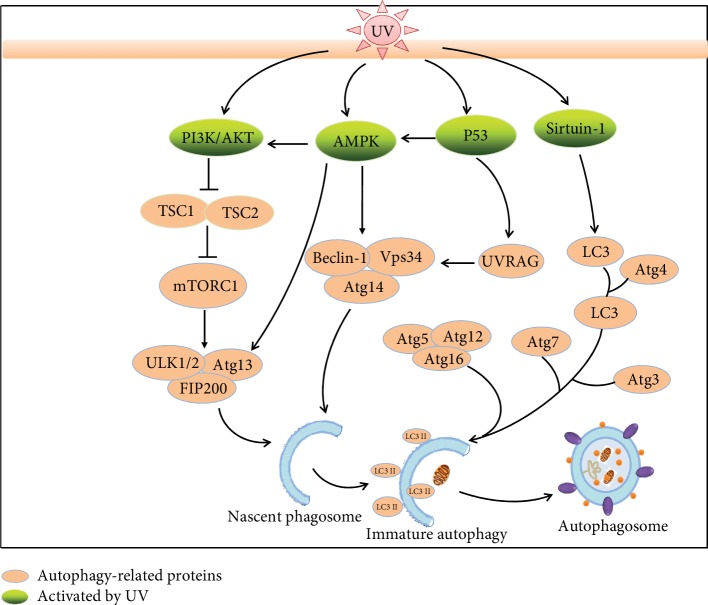
UV radiation modulates the autophagy process via multiple signaling pathways (AMPK, PI3K/Akt, p53, and sirtuin-1). Solar UV irradiation promotes PI3K/Akt activation to inhibit TSC1/2. These signals converge on mTORC1 (mTOR, RAPTOR, and mLST8), which coordinately modifies the ULK complex to affect early steps of the autophagosome process. UV exposure also affects the signaling pathways through the mTOR-AMPK axis by activating ULK1/2 and promoting preautophagosomal structure formation. The nascent phagosome is subsequently modified by a complex of Beclin-1, ATG14, and Vps34, to form the isolation membrane structures. The expansion of the latter complex is associated with two ubiquitin-like reactions involving Atg7, Atg5, Atg12, Atg16, and Atg3 and ultimately conjugates phosphatidylethanolamine (PE) to LC3. UV activates signaling through the AMPK-Beclin-1/Vps34 complex or the p53-UVRAG-Beclin-1/Vps34 complex, which are involved in the formation of nascent phagosomes. The deacetylase sirtuin-1, a posttranscriptionally acetylating core autophagy protein, is modulated by UV to regulate LC3-I to conjugate LC3 to activate autophagy, and Atgs are involved in the conjugation machinery. LC3-PE conjugation targets LC3 to autophagosomal membranes where it is required for membrane expansion and cargo sequestration. Finally, the autophagosome is sealed and the sequestered cargo is delivered to the lysosome through autophagosome-lysosome fusion. PI3K/Akt: phosphatidylinositol 3-kinase/protein kinase B; MAPK: mitogen-activated protein kinase; mTOR: mammalian target of rapamycin; p53: tumor protein p53: sirtuin-1: silent mating type information regulation 2 homolog; PE: phosphatidylethanolamine; ULK: Unc-51-like autophagy-inhibiting kinase; UVRAG: UV resistance-associated gene; TSC: tuberous sclerosis complex; LC3: microtubule-associated protein 1A/1B light chain 3; FIP200: 200 kDa family-interacting protein.

Solar UV exposure is a crucial part of environmental stress that affects skin tissue damage. Exposure to solar UV radiation induces ROS to activate some cell surface receptors, e.g., epidermal growth factor receptor (EGFR) and tumor necrosis factor receptor (TNFR), and to initiate cell survival or apoptosis-associated signaling cascades [63, 64]. Solar UV radiation activates one of the serine/threonine protein kinase family proteins and is associated with cellular signaling, i.e., the mitogen-activated protein kinase (MAPK) pathway. In general, the MAPK pathways comprise three diverse pathways, viz., c-Jun NH2-terminal kinase (JNK), p38 MAPK (p38 kinase), and extracellular signal-regulated kinase (ERK) pathways [65]. The ERK cascade induces cell proliferation and promotes cell survival, while the other two pathways (JNK and p38 kinase) provide protection and proapoptotic effects, respectively [66, 67]. Each of the serine/threonine protein kinase family activates a different stimulus or cellular stress by targeting specific intracellular proteins. Normally, ERK activation is induced by UVA-mediated ROS, but JNK is mainly activated by UVC, while p38 kinases could be activated by all UV wavelengths (including UVA, UVB, and UVC) to modify DNA damage response [68, 69]. In addition, solar UV radiation can trigger the p53 pathway activity via induction of p53 upregulated modulator of apoptosis (PUMA) and phorbol-12-myristate-13-acetate-induced protein 1 (PMAIP1 or NOXA), resulting in Bcl-2 inhibition and thereby suppressing apoptosis progression [63, 70]. Thus, solar UV as an oxidative agent modulates signal transduction pathways in the cellular response (Figure 3).

## 5. UV-Induced Signaling Pathways and Modulation of Autophagy

Diverse signaling networks are involved in regulating autophagy, and two major kinases mTOR (mechanistic target of rapamycin) and AMPK (AMP-activated protein kinase) are linked to aging and lifespan regulation (Figure 3). mTOR, as a negative regulator of autophagy, integrates signals from nutrients and stress to control cellular growth and metabolism. When stimulated by environmental stress, such as solar UV, mTOR phosphorylates the unc-51-like autophagy-inhibiting kinase (ULK) 1 and activates it to form a complex with Atg13 and FIP200, thereby inhibiting autophagy. Inversely, inhibition of mTOR promotes autophagy [71]. In contrast to mTOR, AMPK activation induces the autophagy process and AMPK itself is activated by energy stress, which is sensed through an increase in the AMP/ATP ratio. Upon activation, AMPK stimulates autophagy through multiple mechanisms. First, it phosphorylates and activates ULK1 and Beclin-1-VPS34 to promote the early steps of autophagosome induction. Second, AMPK inhibits mTOR by phosphorylating and inhibiting RAPTOR (regulatory-associated protein of mTOR), an important adaptor for mTOR kinase activity. Finally, AMPK stimulates tuberous sclerosis complex (TSC) 1-TSC2 complex activity, which inhibits mTOR. Oxidative stress, such as UV exposure, notably UVB-induced the phosphorylation of AMPK and increased levels of the AMPK downstream target genes acetyl-CoA-carboxylase (ACC) and ULK1 in wild-type MEF cells. AMPK activation by UVB increases LC3-II levels and autophagic flux, whereas an AMPK knockdown significantly reduces LC3-II levels [5]. Serine/threonine-specific protein kinase B (PKB or Akt) inhibits autophagy through mTOR activation, and PI3K/Akt activation is induced by UVB radiation [72, 73]. The UV resistance-associated gene (UVRAG) generally acts as an autophagy promoter, and inhibition of UVRAG levels causes autophagy to activate suppression [74]. Upon stabilization by UVB, autophagy initiation activates the transcription of AMPK, Sesn2, TSC2, and UVRAG [6]. In addition, p53 accumulation is increased in mammalian cells following UV radiation [75]. UV-mediated signaling pathways involved in autophagy are depicted in (Figure 3). Together, autophagy was modulated by various UV-mediated signaling pathways.

## 6. UV Directs the Regulation of Autophagy and Aging-Related Transcriptional Factors

### 6.1. Mammalian Target of Rapamycin (mTOR)

The mTOR exists in two different enzymatic isoforms, i.e., mTORC1 and mTORC2. They are primary negative regulators of autophagy in almost all eukaryotic organisms. These enzymatic isoforms regulate various types of substrates, such as the Akt/mTOR pathway, which is an important mediator of natural aging [76]. Young et al. and Young and Narita identified that mTORC1 activity is suppressed in Ras senescence following the mitotic phase and that its inactivation coincides with the initiation of autophagy during the transition phase [77, 78]. Recently, it was reported that solar UV exposure, mainly in the 290-320 nm region (UVB), activates the mTORC2/Akt/IKK*α* signaling cascade in human HaCaT keratinocytes, while suppression of mTORC2 inhibits UVB-mediated activation of NF-*κ*B via downregulation of Akt/IKK signaling. In addition, the UV-mediated induction of mTORC2 signaling in skin aging is directly linked to stimulation of NF-*κ*B [79]. Various stressors, particularly starvation, inhibit the activation of mTOR while promoting the expression of ULK1, ULK2, and Atg13 leads to starvation-mediated initiation of autophagy (Table 1) [40].

### 6.2. Silent Mating Type Information Regulation 2 Homologs (Sirtuins or SIRTs)

The NAD(+)-dependent sirtuin enzymes are well-known modulators of aging, enhancing the lifespan of organisms [80] due to their extensive biological roles in metabolic control, cell death and survival, gene repression, repair of damaged DNA, morphogenesis, natural aging, and inflammation [81]. During cellular senescence, the expression of SIRT-1 decreases, while transgenic overexpression of SIRT-1 induces cell proliferation to inhibit senescence [37, 82]. Recently, seven different sirtuins (i.e., SIRT-1 to SIRT-7) have been reported in humans, and the levels of their expressions have also been well documented in human epidermal and dermal cells [83]. SIRT-1 expression has been reported to decline following UV exposure in skin keratinocytes [84]. It is specifically localized in the cell nucleus and sometimes in the cytoplasm. It has been reported that SIRT-1's cytoplasmic location is highly effective as the initiator of autophagy. Resveratrol mediates autophagy in enucleated cells through SIRT-1, suggesting that SIRT-1 initiates autophagy *via* a nonnuclear process [85]. Accordingly, SIRT-1 deacetylates the autophagy-related genes Atg5, Atg7, and Atg8/LC3 and the transcription factor forkhead box 3, which stimulates the expression of proautophagic genes [86]. Consequently, SIRTs induce cellular senescence in keratinocytes and particularly SIRT-1, which deacetylates autophagy-linked genes (Table 1).

### 6.3. Forkhead Box Class O (FoxOs)

In mammals, four different FoxO proteins have been isolated, viz., FoxO1 (FoxO1a), FoxO3 (FoxO3a), FoxO4, and FoxO6 [87]. FoxOs are involved in counteracting oxidative stress and cell fate regarding cell apoptosis or senescence. FoxOs are situated downstream of insulin and insulin-like growth factor-1 (IGF-1), which accelerates aging by suppressing FoxOs [88]. Some studies reported that both UVA and UVB irradiation significantly decrease the expression of FoxO1a and type I collagen (COL1A) mRNA in fibroblasts, while MMP-1 and MMP-2 expression levels are increased [89]. FoxO is homologous to the *Caenorhabditis elegans* (*C. elegans*) transcription factor abnormal dauer formation-16 (DAF-16), which is a downstream gene of the insulin receptor DAF-2 and is actively involved in the regulation of organism lifespan [90]. In various other nematodes, DAF-2 mutants (DAF-2 encodes a hormone receptor similar to the insulin and IGF-1 receptor) have been inactivated by either DAF-16 or autophagy inhibition using genetic or molecular approaches. However, genetic modifications for overexpressing DAF-16/FoxO proteins might enhance autophagy in *C*. *elegans*. The latter provides evidence for the important role of FoxOs in autophagy. Furthermore, FoxO3 can enhance autophagy via regulating the glutamine metabolism. The targeting or selective activation of FoxOs (FoxO3, FoxO4) leads to an increase in glutamine production. The activation of FoxOs possibly directs mTOR inhibition by inhibiting translocation of FoxOs into the lysosomal membranes in a glutamine synthetase-dependent manner, which consequently enhances autophagy progression [91]. In neonatal rat cardiac myocytes, upregulation of either SIRT-1 or FoxO1 is sufficient for autophagic flux induction, whereas both are required for glucose deprivation-induced autophagy (Table 1) [92]. Taken together, autophagy-related factors appear to be involved in UV-mediated photoaging.

## 7. UV Modulated Oxidative Stress-Related Factors

### 7.1. Peroxisome Proliferation-Activated Receptor *δ* (PPAR*δ*)

The ligand-inducible transcription factor PPAR*δ* has been reported to regulate diverse biological phenomena to maintain homeostasis within skin tissues. PPAR*δ* and its specific ligand, GW501516, are activated in human dermal fibroblasts (HDFs) that markedly decrease UVB-induced expression of MMP-1 and ROS generation. PPAR*δ*-driven inhibition of MMP-1 expression is linked with the recovery of original COL-I and COL-III levels, which is mainly due to the prevention of photoaging and restoration of skin integrity [93]. GW501516 treatment also upregulates two well-known autophagy-related markers (Beclin-1 and LC3-II), and PPAR*β*/*δ*-knockout mice show a sharp drop in autophagic marker levels (Table 1) [94].

### 7.2. Heat Shock Protein 70 (HSP70)

HSP70 is a well-known heat shock protein and is normally expressed under certain stresses, such as heat stressors or exposure to heavy metals. HSPs play a crucial role in controlling 3-dimensional protein folding and removing damaged proteins and cellular inclusions [95]. HSP70 plays a critical role in numerous neurodegenerative diseases that are often linked to aging, and treatment with exogenous recombinant human Hsp70 (eHsp70) extends the lifespan of aged mice [96]. During continuous but intermittent UVB exposure, HSP70 transgenic animal models show a slight drop in skin elasticity and epidermal hyperplasia, which is thought to be due to low doses of UVB and the related low production of ROS. This leads to induction of apoptosis in fibroblasts while reducing the infiltration of neutrophils and macrophages within the skin tissue [97]. In chaperone-induced autophagy, cytoplasmic proteins with a clearly exposed pentapeptide motif (KFERQ) are the main targets of HSPA8/HSC70 (heat shock 70 kDa protein 8). Following recognition of their exact motif by HSPA8 and subsequent binding with lysosomal-linked membrane protein 2A (LAMP2A), target proteins undergo unfolding and finally translocate within the lysosomal lumen for their final degradation [98]. It is suggested that the proteasome shuttle factor UBQLN2 identifies client-bound Hsp70 and links it to the proteasome for degradation of accumulated and misfolded proteins in the mouse brain [99] (Table 1).

### 7.3. NF-E2-Related Factor 2 (Nrf2)

Nrf2 is a member of the NF-E2 family of basic leucine zipper transcription factors, and its cytoplasmic inhibitor Kelch-like ECH-associated protein 1 (Keap1) is a major protein that coordinates at the transcriptional level to induce or regulate the expression of different antioxidant enzymes. Under homoeostatic conditions, Keap1 usually keeps Nrf2 tightly bound within the cytoplasm. Upon stimulation (UV or H_2_O_2_), mainly via potent ROS, the Nrf2-Keap1 protein complex is disrupted, and Nrf2 rapidly translocates into the nucleus to target specific genes via heterodimeric combinations with a small Maf protein [100]. UVA irradiation is mainly involved in Nrf2 nuclear translocation and accumulation; hence, it can modulate the downstream effectors [49]. Our previous studies have also suggested that UVA irradiation increases the expression of Nrf2 and its target gene product, HO-1, in human skin fibroblasts [101]. It was reported that UVB irradiation of Nrf2^−/−^ mice accelerates skin photoaging [102]. Furthermore, Kubben and colleagues revealed that repression of the Nrf2-mediated antioxidant response is a critical contributor to premature aging [103]. An Nrf2 knockout in embryonic fibroblasts exhibits reduced expression of autophagic genes, which were rescued by an Nrf2-expressing lentivirus and impaired autophagy flux following exposure to H_2_O_2_. On the other hand, Nrf2 regulates autophagy-associated gene (p62, ULK1, and Atg5) expression in a mouse model of Alzheimer's disease [104]. Meanwhile, p62 interacts with Keap1 at the Nrf2-binding site, and any overexpression or deficiency of p62 in autophagy competes with the interaction between Nrf2 and Keap1, resulting in stabilization of Nrf2 and activation of its downstream targets. This finding indicates that various pathological conditions are linked with excessive accumulation of p62, which potentiates Nrf2 and delineates unexpected functions of selective autophagy by regulating the expression of cellular defense enzymes at the transcriptional level (Table 1) [105]. Taken together, Nrf2 activation by UV appears to be associated with autophagy.

### 7.4. Heme Oxygenase (HO) System

HO-1 is one of the main stress response proteins induced following UVA radiation. To date, two isoforms of the HO system, HO-1 and HO-2, have been defined. The HO system is reported to degrade heme molecules into carbon monoxide (CO), free cellular ferrous iron (Fe), and biliverdin [106]. Both of these HO isoforms share approximately 45% amino acid sequence similarity, with HO-2 mainly present in a constitutive form and HO-1 present in inducible forms within the skin cell [107]. HO is evolutionarily conserved in the human genome, and HO-1 (approximately 32 kDa) and HO-2 (approximately 36 kDa) are encoded by the HMOX1 and HMOX2 genes, respectively. It was found that HO-1 has high anti-inflammatory and antiapoptotic properties that are vital in preventing inflammation-related cell signaling [108]. On the other hand, disturbances in the HO-1 level are associated with some age-dependent disorder pathogenesis, including neurodegeneration, macular degeneration, and cancer [109]. The expression of HO-1 varies according to tissue type. The highest expression in fibroblasts occurs following exposure to ROS-mediated oxidative stress, while epidermal keratinocytes have low levels of HO-1. In contrast, their constitutive expression of HO-2 is high [110] in keratinocytes. The increased expression of HO under oxidative stress conditions is likely to relate to its cytoprotective role [111, 112]. Additionally, UVB effects on skin are well documented, and UVB barely induces HO-1, possibly due to its low production of ROS [113]. It was also found that lipopolysaccharide (LPS) mediates autophagy signals in macrophages via Toll-like receptor 4 (TLR4). This process is dependent on the HO-1 signaling pathway in macrophages [114]. A large amount of data reveals that HO-1 and autophagy are both upregulated in liver cells after cervical ligation and puncture in C57BL/6 mice or in primary mouse hepatocytes upon exposure to LPS. The pharmacological prevention of HO-1 expression through either tin protoporphyrin or knockdown procedures also reduces the production of autophagic signaling in such models and causes additional hepatocellular injury and apoptotic death (Table 1) [115].

### 7.5. Nuclear Factor-Kappa B (NF-*κ*B)

NF-*κ*B is a well-known transcription factor activated by UV light exposure [116]. It is an acute inducer that produces cell responses to inflammation-producing cytokines, signal creation, various types of pathogens, and cell stresses. In resting cells, NF-*κ*B remains silent in the cytoplasm through stoichiometric linkage with its inhibitory proteins, i.e., I*κ*Bs. The NF-*κ*B pathway is involved in accelerating the progression of aging [117], and NF-*κ*B attenuates oxidative stress and DNA damage and delays cellular senescence [118]. A low dose of UVB irradiation activates AP-1 and NF-*κ*B, resulting in elevated MMP expression that degrades collagen and elastin and thus disrupts the integrity of skin tissue, leading to solar scars that accumulate over a lifetime due to repeated and continuous low doses of solar light exposure in photoaging [119]. This dormant NF-*κ*B pool is activated by certain inflammatory triggers that activate the I*κ*B kinase (IKK) complexes and allow targeted phosphorylation of the canonical I*κ*B proteins (I*κ*B*α*, I*κ*B-*β*, and I*κ*B-*ε*), targeting them for ubiquitination and proteasomal degradation. As a result, NF-*κ*B sequentially gathers within the nucleus and activates associated genes [120]. UV radiation activates NF-*κ*B primarily in two segments, i.e., a DNA damage-independent stage [116, 121] and a DNA damage-dependent stage (>24 hours) [122]. The late stage of NF-*κ*B activation has been well studied and involves activating IKK by linking with the DNA double-stranded break-activated kinase ataxia telangiectasia mutated (ATM) [123]. Furthermore, a combination of a low concentration (0.2-1 *μ*g/ml) of curcumin and UVA irradiation might induce apoptosis in human skin keratinocytes through enhanced fragmentation of the nucleus, discharge of cytochrome c from mitochondria, initiation of the caspase cascade (Casp-8 and Casp-9), and disruption of NF-*κ*B cascades [124]. Previously, Reelfs and coworkers reported that UVA irradiation-activated proinflammatory NF-*κ*B factors are iron-dependent in human skin fibroblasts [125]. Moreover, after exposure to UVA radiation, NF-*κ*B is activated following degradation of its regulatory inhibitory protein (I*κ*B*α*) and via its extended iron-dependent, I*κ*B*α*-independent activation [126]. In various studies, NF-*κ*B has been reported to exert an anti-inflammatory effect by delaying accumulation of the autophagy receptor p62/SQSTM1 and the “NF-*κ*B-p62 mitophagy” pathway is a macrophage-intrinsic regulatory loop that restrains specific proinflammatory processes and arranges a self-limiting host reaction to help restore homeostasis and ultimately repair tissues [127]. In addition, NF-*κ*B RELA cytosolic ubiquitination is stimulated by TLR2 signaling and leads to its degradation through SQSTM1/p62-mediated autophagy, while inhibition of autophagy rescues NF-*κ*B activity and shapes hepatoma-polarized M2 macrophages [128]. Furthermore, inhibition of NF-*κ*B leads to cells becoming sensitive to perturbations in mitochondrial metabolism and autophagy in B cell lymphoma [129] (Table 1). NF-*κ*B therefore modulates photodamage and photoaging mediated by UV and is also involved in mitophagy and macrophagy.

## 8. Conclusion

UV exposure is a major factor that induces photoaging by elevating the level of oxidized lipid and metabolite aggregate levels. Loss of autophagy leads to diverse cellular dysfunctions that exacerbate the aging process, while elevated autophagy generally promotes cellular homeostasis, prolongs lifespan, and improves health life quality. Autophagy induction increases metabolite adduct degradation by UV irradiation-induced ROS which in turn lead to inhibition of photoaging. In contrast, a decrease in autophagy is likely to promote skin photoaging and the promotion of UV-induced damage. The current approach to prevent photoaging mainly relies on the avoidance of sunlight exposure to the skin. Antioxidants and DNA repair-related enzymes can be added as ingredients to sunscreens to enhance their photoprotective potential against sunlight exposure to the skin. While much progress has been made in combatting photoaging triggered by UV, the role of autophagy in resisting photoaging yet remains to be elucidated. Autophagy plays a critical role in UV-induced apoptosis, DNA damage repair, oxidized lipid removal, and so on. Autophagy may therefore be considered a new pathway to prevent photoaging and skin cancer. The understanding of the mechanisms underlying the switch between autophagy and photoaging provides valuable insights into UV-associated diseases and therapeutic methods. This in turn should offer a molecular platform for autophagy-targeted treatment to slow down aging-related chronic diseases including photoaging and other UV-induced oxidative disorders such as skin cancer.

## Figures and Tables

**Table 1:** Activation of UVR-responsive genes and their association with autophagy.

Gene	Relation to aging	Type of UV radiation	Relation to autophagy	References
mTOR (mTORC1 and mTORC2)	mTORC1 suppresses RAS-induced senescence, and mTORC2 induces skin aging through activation of NF-*κ*B cascade	UVB activates the mTORC2/Akt/IKK*α* pathway	mTOR negatively regulates autophagy via Atg13, ULK1, and ULK2	[73–77]
Sirtuins	Sirtuins modulate lifespan, while SIRT-1 inhibits senescence	UV exposure deceases SIRT-1 in skin keratinocytes	SIRT-1 induces autophagy through deacetylation and activation of autophagy-related genes ATG5, ATG7, and ATG8/LC3.	[40, 78–83]
FoxOs	FoxOs are regulated by IGF-1, while IGF-1 induces aging	FoxO3 induces autophagy by glutamine metabolism; FoxO1 overexpression induces autophagic flux formation	UVA and UVB radiation decreases FoxO1 expression in fibroblasts	[84–89]
PPAR*δ*	PPAR*δ* prevents photoaging by the inhibition of MMP-1	UVB attenuates PPAR*δ* through the induction of MMP-1 secretion	PPAR*δ* activation induces autophagy marker Beclin-1 and LC3 expression	[90, 91]
Hsp70	eHsp70 treatment prolongs lifespan of mice	UVB chronic exposure induces ROS-mediated apoptosis and decreases macrophagy	HSPA8/HSC70 plays an important role in chaperone-mediated autophagy. Hsp70 links to the proteasome shuttle factor UBQLN2 to degrade misfolded proteins	[92–96]
Nrf2	Nrf2 deficiency in mice following UVB irradiation promotes mouse photoaging; repression of the Nrf2-mediated antioxidative response contributes to premature aging	UVA exposure increases Nrf2 expression in fibroblasts. UVB induces mouse photoaging by Nrf2 depletion	Nrf2 knockout reduces expression of autophagic genes in embryo fibroblasts	[97, 98]
HO-1	Disturbances in HO-1 level are associated with age-dependent disorder pathogenesis	Both UVA and UVB induce detoxifying enzyme HO-1 expression	HO-1 and autophagy are upregulated by LPS in primary mouse hepatocytes; pharmacological knockdown or inhibition of HO-1 prevents autophagy	[99–108]
NF-*κ*B	NF-*κ*B pathway is involved in progression of aging, and NF-*κ*B inhibition attenuates oxidative stress, DNA damage, and delayed cellular senescence	UV activates NF-*κ*B to form two phases Curcumin combined with UVA induces apoptosis by inhibition of NF-*κ*B activity, and UVA exposure activates NF-*κ*B	Inhibition of NF-*κ*B promotes autophagy, while autophagy suppression restores NF-*κ*B activity	[109–125]

## Abbreviations

ACC: Acetyl-CoA carboxylase
Atg7: Ubiquitin-E1-like enzyme
COL1A: Type I collagen
ECM: Extracellular matrix
ERK: Extracellular signal-regulated kinase
ER: Endoplasmic membrane
FAK: Focal adhesion kinase
FIP200: Family-interacting protein of 200 kDa
FoxO: Forkhead box class O
GM-CSF: Granulocyte-macrophage colony stimulatory factor
HDFs: Human dermal fibroblasts
HO: Heme oxygenase
HSP70: Heat shock protein 70
IGF-1: Insulin-like growth factor-1
IL: Interleukin
JNK: c-Jun NH2-terminal kinase
Keap1: Kelch-like ECH-associated protein 1
LAMP2A: Lysosomal-linked membrane protein 2A
LC3: Microtubule-associated protein 1A/1B light chain 3
MAPK: Mitogen-activated protein kinase
MMPs: Matrix metalloproteinases
mTOR: Mammalian target of rapamycin
NF-*κ*B: Nuclear factor-kappa B
Nrf2: Nuclear factor erythroid-derived 2-like 2
PMAIP1: Phorbol-12-myristate-13-acetate-induced protein 1
PPAR*δ*: Peroxisome proliferation-activated receptor *δ*
PUMA: p53 upregulated modulator of apoptosis
ROS: Reactive oxygen species
Sirtuin: Silent mating type information regulation 2 homolog
TLR4: Toll-like receptor 4
UV: Ultraviolet.

## Additional Points

*Highlights*. (1) UV-mediated ROS generation promotes the progression of the photoaging process. (2) Increased autophagy delays the UV-mediated photoaging, and the inhibition of autophagy enhances the UV-mediated photoaging process. (3) UV-mediated ROS generation activates the signaling pathways responsible for the modulation of the autophagy process. (4) UV directs the regulation of autophagy and aging-related transcriptional factors.
